# Feasibility of structured light Plethysmography (SLP) in patients with coronavirus disease 2019 (COVID-19)

**DOI:** 10.1186/s13019-021-01395-0

**Published:** 2021-03-03

**Authors:** Natalie Simon, Azhar Hussain, Priyanka Kolvekar, Shyam Kolvekar

**Affiliations:** 1 Cambridge University NHS Foundation Trust, Cambridge, UK; 2 Thoracic Surgery, Bart’s Heart Centre, St Bartholomews Hospital, London, UK; 3 University of Warwick, Coventry, UK

**Keywords:** Coronavirus, Chest wall deformity

## Abstract

As a result of the COVID-19 pandemic, most institutions have changed the way patients are assessed or investigated. Using novel non-contact technology, it is possible to continuously monitor the lung function of peri-operative patients undergoing cardiothoracic procedures. Primarily, this results in increased patient surveillance, and therefore, safety. Many centres, globally, are starting to use structured light plethysmography (SLP) technology, providing a non-aerosol generating procedure in place of traditional spirometry. While more evidence is needed, our clinical usage; previous and on-going studies; demonstrate definite potential that SLP is a valuable tool.

Correspondence:

The clinical manifestations of COVID-19 range from mild upper respiratory tract illnesses to progressive severe pneumonia, acute respiratory distress syndrome, multi-organ failure, and death. Measures to control the impact of the virus have affected job security, social contact and challenged health services, medical practices and policies. The health service has been radically mobilised to respond to the acute needs of patients infected with the virus at the same time as delivering scaled-back non-COVID-19 healthcare. In many surgical specialties, the management of perioperative patients has changed, with greater focus on remote triage through virtual consultations. However, pre-operative evaluation of surgical candidates still must happen, in person, at the clinic. One important aspect of pre-operative evaluation prior to thoracic surgery is pulmonary function testing (PFT), traditionally measured by conventional spirometry.

Understandably, concern has been raised that PFT represents a potential avenue for increased COVID-19 transmission due to increased viral dissemination. The healthcare sector is calling upon novel technology to replace PFT with a non-aerosol-generating alternative.

Structured Light Plethysmography (SLP), delivered by PneumaCare Thora-3DI™ systems, has been proposed as a novel, non-contact, non-invasive method of assessing lung function. SLP offers real-time regional respiratory function via movement of the chest wall. This detailed information is then translated into quantifiable pulmonary function outputs. SLP data has been used to optimize non-invasive ventilatory settings. The field has used SLP to successfully differentiate between the breathing patterns of healthy patients and those with COPD by mapping the thoracoabdominal displacement rate and accurately estimating inspiratory and expiratory flow [1]. The ability to continuously measure these parameters may contribute to the safe weaning of patients from ventilatory support. Additionally, we believe that SLP technology will allow clinicians to obtain measurements of breathing patterns that are closer to ecological conditions than those derived from spirometry measurements.

Our centre is currently trialling SLP in the work-up of pre-operative cardiothoracic patients. We have so far enrolled 20 patients in our pilot study comparing the feasibility of SLP measurements as parameters indicative for lung function. Our initial results demonstrated a correlation between the IE50_SLP_ (inspiratory to expiratory flow at 50% tidal volume) measurement and the forced expiratory volume in the first second of expiration (*p* = 0.021, r = − 0.62). We feel it is intuitive to use and, therefore, does not require trained technicians, resulting in increased patient throughput and lower costs to the department. Care efficiency also improves as the time-per-appointment is reduced, benefitting patient wait times and increasing satisfaction. Importantly, SLP offers clinicians continuous measurement of mechanical chest wall displacement, a surrogate marker for fatigability and neuromuscular strength.

For the aforementioned reasons, we feel that SLP is a viable alternative to spirometry, especially in the current pandemic. Spirometry remains the gold-standard PFT, however, and the field would certainly benefit from studies evaluating the sensitivity, specificity and clinical validity of SLP in impairment detection, against gold-standard PFT.

## Data Availability

Not applicable.

## Abbreviations

COVID-19: Coronavirus disease
SLP: Structure light plethysmography
PFT: Pulmonary function tests
COPD: Chronic obstructive pulmonary disease
